# Upregulation of calcium-sensing receptor and mitogen-activated protein kinase signalling in the regulation of growth and differentiation in colon carcinoma

**DOI:** 10.1038/sj.bjc.6602852

**Published:** 2005-11-08

**Authors:** N Bhagavathula, E A Kelley, M Reddy, K C Nerusu, C Leonard, K Fay, S Chakrabarty, J Varani

**Affiliations:** 1 Department of Pathology, University of Michigan Medical School, Ann Arbor, MI 48109-0602, USA; 2 Southern Illinois University Cancer Institute, Springfield, IL 62794-9677, USA

**Keywords:** colon carcinoma, calcium sensing receptor, mitogen-activated protein kinase, E-cadherin, differentiation, calcium, growth arrest

## Abstract

In the present study, we demonstrate that Ca^2+^-induced growth inhibition and induction of differentiation in a line of human colon carcinoma cells (CBS) is dependent on mitogen-activated protein (MAP) kinase signaling and is associated with upregulation of extracellular calcium-sensing receptor (CaSR) expression. When CBS cells were grown in Ca^2+^-free medium and then switched to medium supplemented with 1.4 mM Ca^2+^, proliferation was reduced and morphologic features of differentiation were expressed. E-cadherin, which was minimally expressed in nonsupplemented medium, was rapidly induced in response to Ca^2+^ stimulation. Sustained activation of the extracellular signal-regulated kinase (ERK) occured in Ca^2+^-supplemented medium. When an inhibitor of ERK activation (10 *μ*M U0126) was included in the Ca^2+^-supplemented culture medium, ERK-activation did not occur. Concomitantly, E-cadherin was not induced, cell proliferation remained high and differentiation was not observed. The same level of Ca^2+^ supplementation that induced MAP kinase activation also stimulated CaSR upregulation in CBS cells. A clonal isolate of the CBS line that did not upregulate CaSR expression in response to extracellular Ca^2+^ was isolated from the parent cells. This isolate failed to produce E-cadherin or undergo growth inhibition/induction of differentiation when exposed to Ca^2+^ in the culture medium. However, ERK-activation occurred as efficiently in this isolate as in parent CBS cells or in a cloned isolate that underwent growth reduction and differentiation in response to Ca^2+^ stimulation. Together, these data indicate that CaSR upregulation and MAP kinase signalling are both intermediates in the control of colon carcinoma cell growth and differentiation. They appear to function, at least in part, independently of one another.

Calcium (Ca^2+^) has growth-inhibiting and differentiation-promoting activities for a variety of normal and malignant epithelial cells, including cells of the gastrointestinal tract (Lamprecht and Lipkin, 2003). Compelling experimental and epidemiological evidence suggests an inverse relationship between dietary intake of Ca^2+^ and risk of colorectal cancer (Sellers *et al*, 1998; Baron *et al*, 1999; Lipkin, 1999; Kampman *et al*, 2000; Wargovich *et al*, 2000; Wu *et al*, 2002; McCullough *et al*, 2003). Although such findings strongly imply a role for extracellular Ca^2+^ in regulating growth and differentiation in colon epithelium, the mechanism(s) by which this occur(s) is/are poorly understood.

Recent studies suggest that the extracellular calcium-sensing receptor (CaSR) is involved in the regulation of colon carcinoma cell growth and differentiation (Chakrabarty *et al*, 2003, 2005). The CaSR is expressed in a number of human colon carcinoma cell lines derived from moderately-differentiated tumours. The CaSR promoter has two distinct Ca^2+^-responsive sites and exposure of colon carcinoma cells to extracellular Ca^2+^ upregulates CaSR expression. That CaSR expression might be critical to the growth-reduction and differentiation-induction by Ca^2+^ in these cells is suggested by the finding that gadolinium (Gd^3+^), an analogue of Ca^2+^ which binds to the CaSR but does not enter cells (Garrett *et al*, 1995; Hebert and Brown, 1995; Rutten *et al*, 1999), is equally efficient as Ca^2+^ in reducing growth and inducing differentiation (Chakrabarty *et al*, 2003).

The present studies further our efforts to understand mechanisms of growth regulation in colon epithelium by extracellular Ca^2+^. Here, we show that the extracellular signal-regulated kinase (ERK) cascade of the mitogen-activated protein (MAP) kinase family is activated in a sustained fashion by extracellular Ca^2+^ in a line of colon carcinoma cells (CBS), concomitant with growth suppression and induction of differentiation. Interference with ERK activation using the inhibitor U0126 prevents Ca^2+^-induced alterations in growth and differentiation. A CBS clone that fails to upregulate CaSR in response to Ca^2+^ also fails to demonstrate growth reduction or induction of differentiation under the same conditions. However, when the CaSR-defective cells are exposed to extracellular Ca^2+^, ERK activation occurs as efficiently as in the parent cells. Taken together, these data indicate that both CaSR upregulation and MAP kinase signalling are critical intermediates in the control of colon carcinoma cell growth and differentiation. The two events appear to be (at least in part) independent of each other.

## MATERIALS AND METHODS

### Reagents

Reagents used in these studies included antibodies to the total and phosphorylated forms of ERK, obtained from Cell Signaling Technologies Inc.; Beverly, MA, USA; antibody to E-cadherin, obtained from Chemicon International Inc., Temecula, CA, USA; antibody to CaSR, obtained from Alpha Diagnostic International, San Antonio, TX, USA; and antibody to *β*-tubulin, obtained from Santa Cruz Biotechnology Inc., Santa Cruz, CA, USA. Signalling inhibitors included U0126 (ERK inhibitor), obtained from Cell Signaling Technologies Inc. (Favata *et al*, 1998); SB203580 (P38 inhibitor), obtained from Calbiochem, San Diego, CA, USA, (Moon *et al*, 2002); SP600125, an anthrapyrazolone inhibitor of the Jun-N-terminal kinase (JNK) obtained from Axxora LLC, San Diego, CA, USA (Bennett *et al*, 2001); and LY294002, an inhibitor of phosphatidylinositol-3-kinaase (PI3K) obtained from Cell Signaling Technologies (Panka *et al*, 2001).

### Cell culture

The moderately-differentiated colon carcinoma cell line, CBS, was used in these studies. This cell line was described in our past reports dealing with CaSR expression and colon carcinoma growth control (Chakrabarty *et al*, 2003, 2005). The cells were maintained in Ca^2+^-free minimal essential medium – Spinner modified (SMEM) (Sigma Chemical Co., St Louis, MO, USA) supplemented with sodium bicarbonate, amino acids, and 5% foetal bovine serum (FBS). Growth was at 37°C in an atmosphere of 95% air and 5% CO_2_. Cells were subcultured by brief exposure to trypsin/EDTA solution.

Random cell cloning was used to obtain several isolates of the CBS line. Briefly, a single-cell suspension of the parent cells was fractionated using a flow cytometer (Facscan, Becton-Dickinson) and one cell per well deposited into wells of a 96-well dish containing 100 *μ*l of CBS-conditioned medium. When individual colonies were of sufficient size, they were subcultured into 25-cm^2^ flasks. When the flasks reached confluence, there were sufficient cells for use in experiments. Cloned isolates were maintained exactly as the parent cells.

### Cell proliferation assay

Cells were plated in 24-well tissue culture dishes at 4 × 10^4^ cells per well using Ca^2+^-free SMEM with 5% FBS as culture medium. After allowing the cells to attach overnight, they were washed twice with Ca^2+^-free SMEM without FBS and then incubated in Ca^2+^-free SMEM with 5% dialysed FBS. Different amounts of Ca^2+^ were then added to individual wells as indicated in the Results. Proliferation was measured by releasing the cells with trypsin/EDTA and enumerating them using a particle counter (Coulter Electronics, Hialeah, FL, USA). For dose–response studies, cells were harvested on day-3. For time-course studies, cell counts were made on days 1–4.

### Immunohistochemistry

Immunoperoxidase staining for E-cadherin was performed as described previously using formalin-fixed CBS cells (Sheinin *et al*, 2000; Chakrabarty *et al*, 2003). The immunoperoxidase reaction product was visualised using diaminobenzidine as the chromogenic substrate. Immunostained sections were lightly counterstained with haematoxylin and examined by light microscopy.

### Preparation of cell lysates and immunoblot analysis

CBS cells were plated at 3 × 10^5^ cells per well in wells of a six-well dish using Ca^2+^-free SMEM with 5% FBS as culture medium. The cells were allowed to attach overnight. The next day, they were washed and then incubated in Ca^2+^-free SMEM with 5% dialysed FBS with or without Ca^2+^ (1.4 mM). After incubation for the desired amount of time, cells were lysed in 1 × cell lysis buffer consisting of 20 mM Tris-HCl (pH 7.4), 2 mM sodium vanadate, 1.0 mM sodium fluoride, 100 mM NaCl, 1% NP-40, 0.5% sodium deoxycholate, 25 *μ*g ml^−1^ each aprotinin, leupeptin and pepstatin, and 2 mM EDTA and EGTA. Lysis was performed at 4°C by scraping the cells into lysis buffer and sonicating the samples. Cell lysates were incubated on ice for 15 min and then cleared by microcentrifugation at 16 000 **g** for 15 min. The supernatant fluids were collected and protein concentration was estimated using the BioRad DC protein assay kit (BioRad, Hercules, CA, USA).

Cell extracts containing equivalent amounts of protein (40 *μ*g of total protein per lane) were examined by Western blotting for E-cadherin, CaSR, and signalling intermediates (total and phosphorylated forms of ERK) using procedures described previously (Bhagavathula *et al*, 2004). Briefly, samples were separated in SDS-PAGE under denaturing and reducing conditions and transferred to nitrocellulose membranes. After blocking with a 5% nonfat milk solution in Tris-buffered saline with 0.1% Tween (TTBS) at 4°C overnight, membranes were incubated for 1 h at room temperature with the desired antibody, diluted 1 : 1000 in 0.5% nonfat milk/0.1% TTBS. Thereafter, the membranes were washed with TTBS and bound antibody detected using the Phototope-HRP Western blot detection kit (Cell Signaling Technologies Inc.). Images were scanned, digitised and quantified using NIH image analysis software.

### Statistical analysis

Data from studies with multiple experiments and multiple samples per data point were expressed as mean±s.e. Data from studies with multiple experiments and one sample per data point in each experiment (Western blot experiments) were expressed as mean±s.d. For experiments in which multiple groups were included, statistical analyses were carried out by ANOVA followed by paired group comparisons. For experiments in which two groups were compared to one another, the Student's *t*-test was used. *P*<0.05 was considered statistically significant.

## RESULTS

### Effect of extracellular Ca^2+^ on proliferation of CBS colon cancer cells

**Figure 1:**
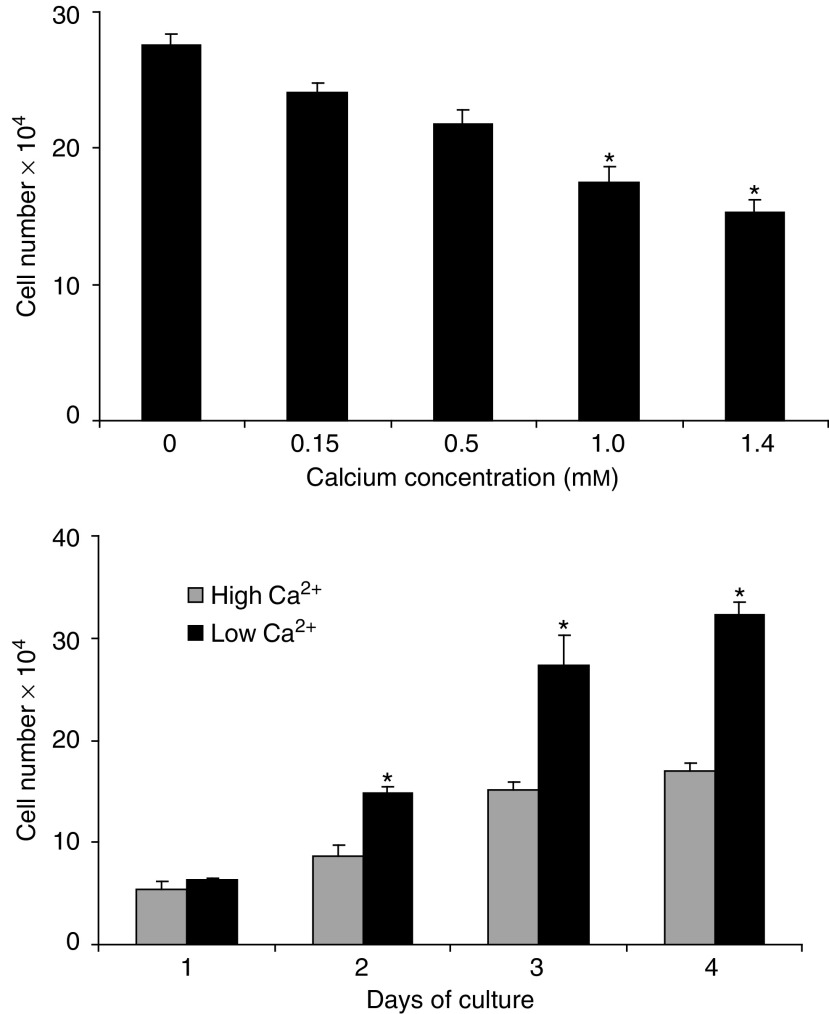
Effect of extracellular Ca^2+^ on proliferation of CBS colon cancer cells. *Upper panel*: dose–response. *Lower panel*: time–response. Values shown are means and s.d. based on triplicate samples in a single experiment. Statistical significance was determined by ANOVA, followed by paired group comparisons or by Student's *t*-test. ^*^ indicates differences from control group (no Ca^2+^ supplementation) at the *P*<0.05 level. Both proliferation studies were conducted five times with consistent results.

**Figure 2:**
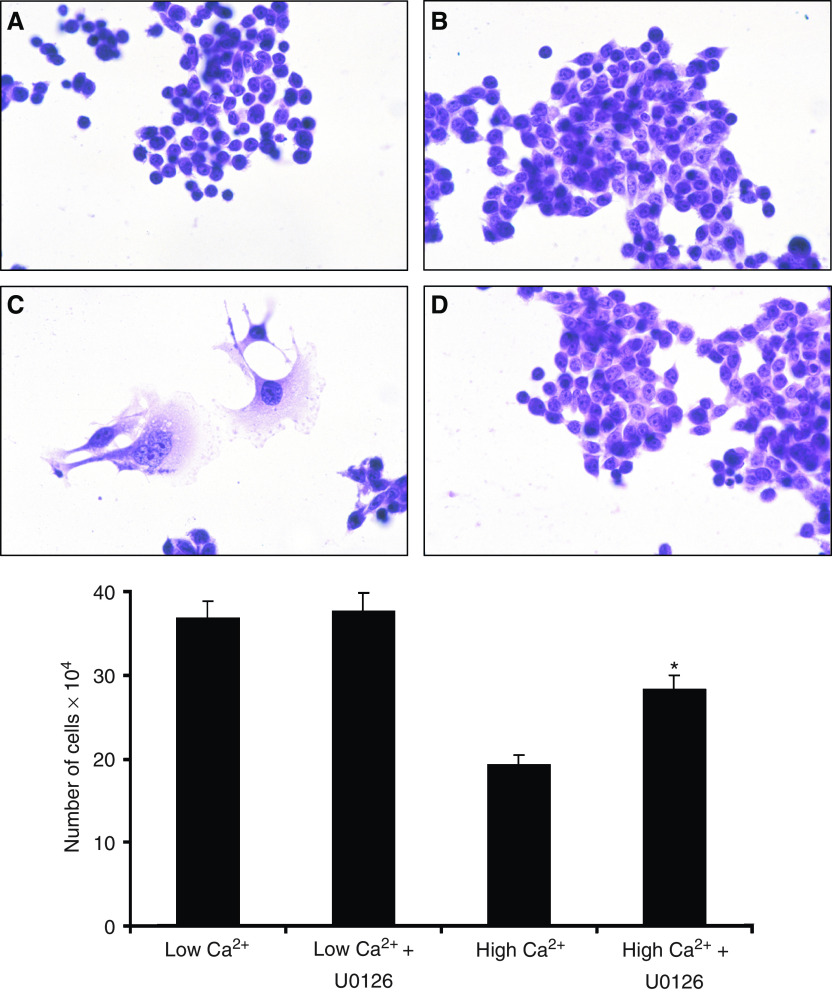
Effect of U0126 on extracellular Ca^2+^-mediated changes in CBS cell growth and differentiation. *Upper panel*: morphological appearance of CBS cells grown for 3 days in Ca^2+^-free medium with or without 10 *μ*M U0126 (upper left and upper right respectively) or in medium supplemented with 1.4 mM Ca^2+^ with or without 10 *μ*M U0126 (lower left and lower right). *Lower panel*: CBS cell proliferation under the same conditions. Values shown are means and s.d. based on triplicate samples in a single experiment. Statistical significance was determined by ANOVA, followed by paired group comparisons. ^*^ indicates differences from high-Ca^2+^ group (1.4 mM Ca^2+^ supplementation) at the *P*<0.05 level. The study was conducted five times with consistent results.

Initially, the effects of different extracellular Ca^2+^ levels on proliferation of CBS cells was studied. As shown in Figure 1 (upper panel) inhibition of CBS cell proliferation was dose-dependent between 0 and 1.4 mM extracellular Ca^2+^. Growth was inhibited by approximately 45% in the presence of 1.4 mM Ca^2+^. As shown in the lower panel of Figure 1, minimal (not statistically significant) inhibition of growth was observed 1 day after shifting the cells to Ca^2+^ (1.4 mM)-supplemented medium. Statistically significant inhibition was observed on days 2–4. A change in the morphological appearance of the cells accompanied Ca^2+^-induced growth inhibition. Whereas CBS cells grew in small, grape-like clusters under low-Ca^2+^ conditions, they became flattened in the presence of Ca^2+^-supplemented medium (see Figure 2, upper panel for example).

### Ca^2+^-mediated changes in CBS cell proliferation: dependence on ERK-activation

Following the observation of growth inhibition with increasing concentrations of exogenous Ca^2+^, we next sought to identify critical intermediary events leading from Ca^2+^ stimulation to reduced growth. Figure 2 shows effects of the ERK-activation inhibitor U0126 on CBS cell proliferation as well as on morphological appearance in the presence of high (1.4 mM) extracellular Ca^2+^. As compared to cells maintained in SMEM+dialysed FBS (without Ca^2+^ supplementation) throughout the 3-day incubation period, a reduction in cell growth was observed in the presence of 1.4 mM Ca^2+^. Concomitantly, cells maintained in Ca^2+^-supplemented medium underwent a change in morphological appearance (i.e. increased flattening) consistent with differentiation. Both the suppression of proliferation and induction of differentiated features were substantially reversed when U0126 (10 *μ*M) was included in the culture medium throughout the incubation period. These data indicate that Ca^2+^ modulation of growth and differentiation occurs in a MAP kinase-dependent manner.

### Effects of extracellular Ca^2+^ and U0126 on E-cadherin expression

**Figure 3:**
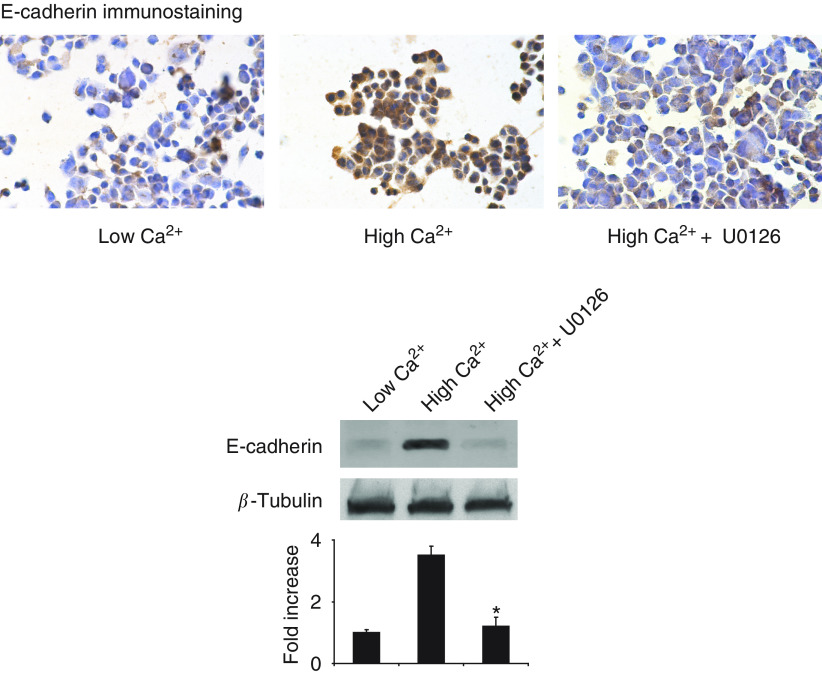
Effect of extracellular Ca^2+^ and U0126 on E-cadherin expression. *Upper panel*: E-cadherin expression by immunostaining of CBS cells grown for three days in nonsupplemented medium (left), in medium supplemented with 1.4 mM Ca^2+^ (centre) and in medium supplemented with 1.4 mM Ca^2+^ and 10 *μ*M U0126 (right). *Lower panel*: E-cadherin expression by Western blotting (same conditions). Quantitative values are based on densitometry scanning of bands in four separate experiments and are means and s.d. Statistical significance was determined by ANOVA, followed by paired group comparisons. ^*^indicates differences from high-Ca^2+^ group (1.4 mM Ca^2+^ supplementation) at the *P*<0.05 level.

The homotypic cell–cell adhesion molecule E-cadherin functions as a tumour suppressor in colon carcinoma cells, and upregulation of E-cadherin expression is associated with the induction of differentiation in these cells (Wang and Chakrabarty, 2001; Van Aken *et al*, 2001). We examined the effect of extracellular Ca^2+^ on the expression of E-cadherin both by immunostaining and immunoblotting techniques. As shown in Figure 3 (upper panel), cells cultured in Ca^2+^-free SMEM plus dialysed FBS for 3 days showed little or no staining for E-cadherin. However, cells grown in the same medium supplemented with 1.4 mM Ca^2+^ showed significant E-cadherin expression. It could be seen that much of the reactivity in the presence of Ca^2+^ was at the cell periphery. When cell lysates were prepared and expression of E-cadherin assessed by Western blotting, a 3.5-fold increase in the expression of E-cadherin was observed in cells grown in Ca^2+^-supplemented medium as compared to the cells grown in Ca^2+^-free medium (Figure 3, lower panel). When U0126 (10 *μ*M) was included in Ca^2+^-supplemented medium, increased E-cadherin expression was largely abolished (Figure 3, upper and lower panels). These observations demonstrate that E-cadherin expression increases in response to extracellular Ca^2+^ in a MAP kinase-dependent manner.

**Figure 4:**
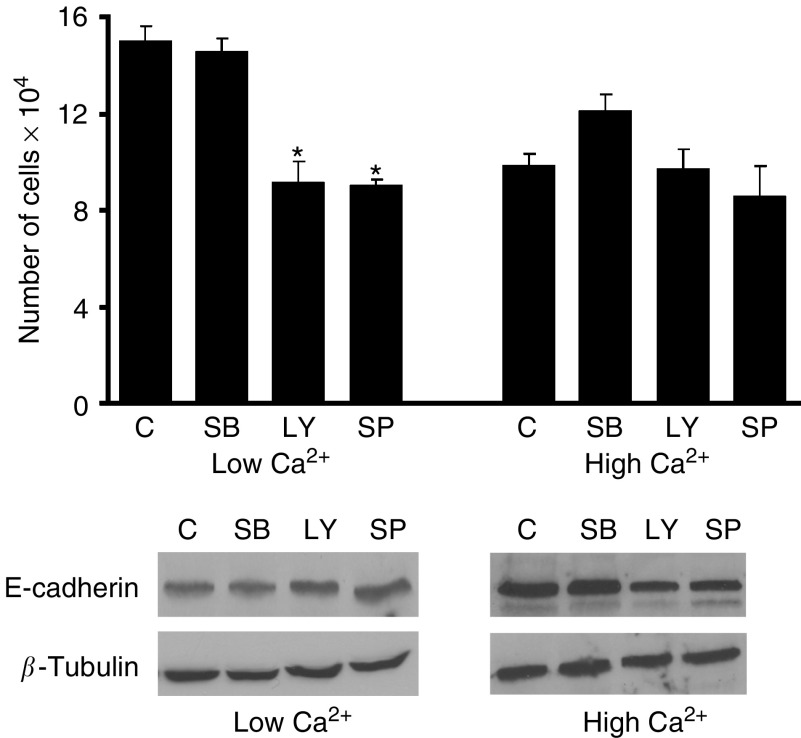
Effect of P38, JNK and PI_3_K inhibitors on CBS cell growth and E-cadherin expression under low-Ca^2+^ and high-Ca^2+^ conditions. *Upper panel*: CBS cell proliferation. Values shown are means and s.d. based on quadruplicate samples in duplicate experiments. Statistical significance was determined by ANOVA, followed by paired group comparisons. ^*^ indicates differences from the respective low-Ca^2+^ or high-Ca^2+^ control groups at the *P*<0.05 level. *Lower panel*: E-cadherin expression and *β*-tubulin expression by Western blotting (same conditions). Western blots were conducted in three separate experiments with consistent results.

In addition to using U0126 to interfere with activation through the ERK pathway, we also utilised inhibitors to block signalling through two other MAP kinase pathways (P38 and JNK) as well as signalling through the PI_3_K/Akt pathway. When CBS cells were treated with the P38 inhibitor, SB203580 (15 *μ*M), there was no effect on cell growth under low-Ca^2+^ conditions and only a modest (not statistically significant) increase in proliferation compared to control cells in high-Ca^2+^ medium (Figure 4). Consistent with this, there was an increased expression of E-cadherin in both control and SB203580-treated cells under high-Ca^2+^ conditions compared to cells in low-Ca^2+^ conditions. In contrast, there was no significant difference in E-cadherin expression between control and SB203580-treated cells (under either condition) (Figure 4). Results with the other two inhibitors were considerably different. Both the JNK inhibitor (SP600125; 0.1 *μ*M) and the PI3K inhibitor (LY294002; 30 *μ*M) strongly inhibited proliferation of CBS cells under low-Ca^2+^ conditions but little effect under high-Ca^2+^ conditions (Figure 4). Consistent with growth-inhibition under low-Ca^2+^ conditions was a slight increase in E-cadherin expression with both agents. Also consistent, the high level of E-cadherin expressed in Ca^2+^-supplemented medium was not further increased by either agent (Figure 4).

### Effects of U0126 on CaSR expression

**Figure 5:**
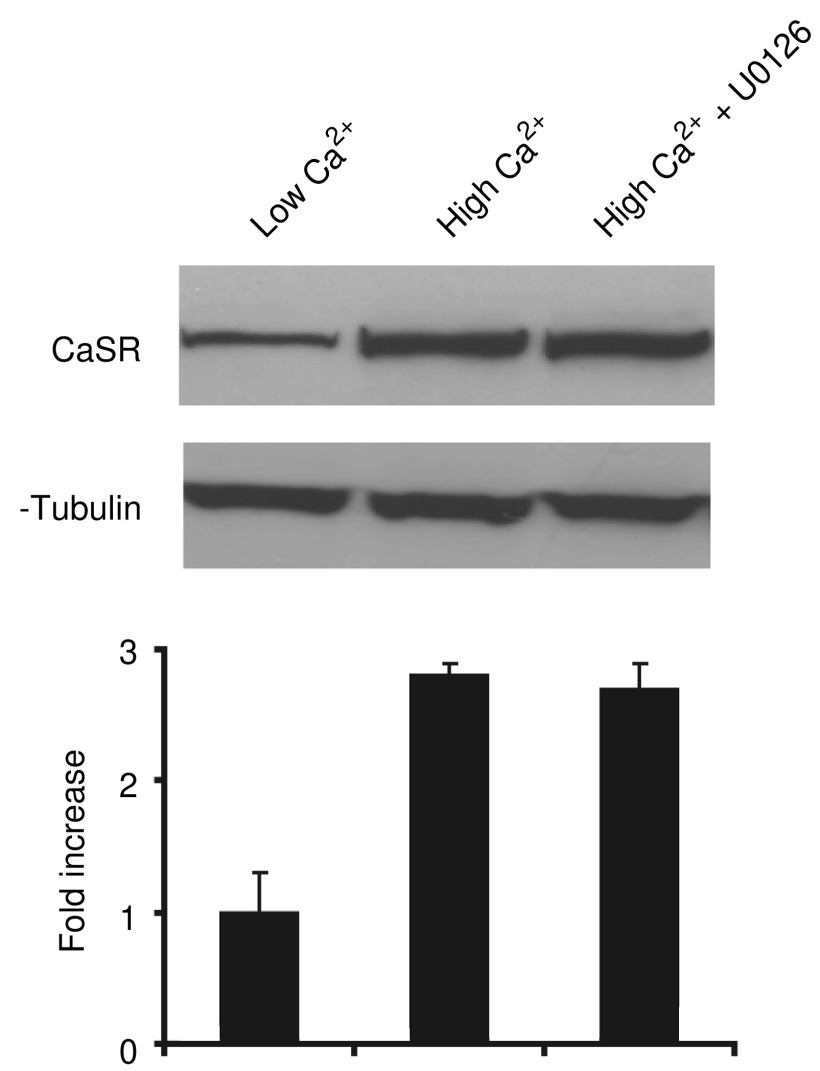
Effect of extracellular Ca^2+^ and U0126 on CaSR expression. Western blotting for CaSR expression in CBS cells grown for three days in nonsupplemented medium (left), in medium supplemented with 1.4 mM Ca^2+^ (centre) and in medium supplemented with 1.4 mM Ca^2+^ and 10 *μ*M U0126 (right). Quantitative values are based on densitometry scanning of bands in four separate experiments and are means and s.d. Statistical significance was determined by ANOVA, followed by paired group comparisons.

Since the effects of extracellular Ca^2+^ on CBS growth and differentiation appeared to involve ERK activation, experiments were conducted to determine if the same treatment influenced CaSR expression. Consistent with our recent studies (Chakrabarty *et al*, 2003, 2005), CaSR was detected in CBS cells maintained for 3 days in Ca^2+^-free medium (SMEM+dialysed FBS), but the expression level was low. When the extracellular Ca^2+^ concentration was increased to 1.4 mM, expression of CaSR was substantially increased (Figure 5). Of interest, upregulation of CaSR expression did not appear to depend on ERK signalling since cells incubated in Ca^2+^-supplemented medium containing 10 *μ*M U0126 showed the same upregulation as cells maintained in the absence of the inhibitor (Figure 5).

### Effect of extracellular Ca^2+^ on expression of phosphorylated-ERK

**Figure 6:**
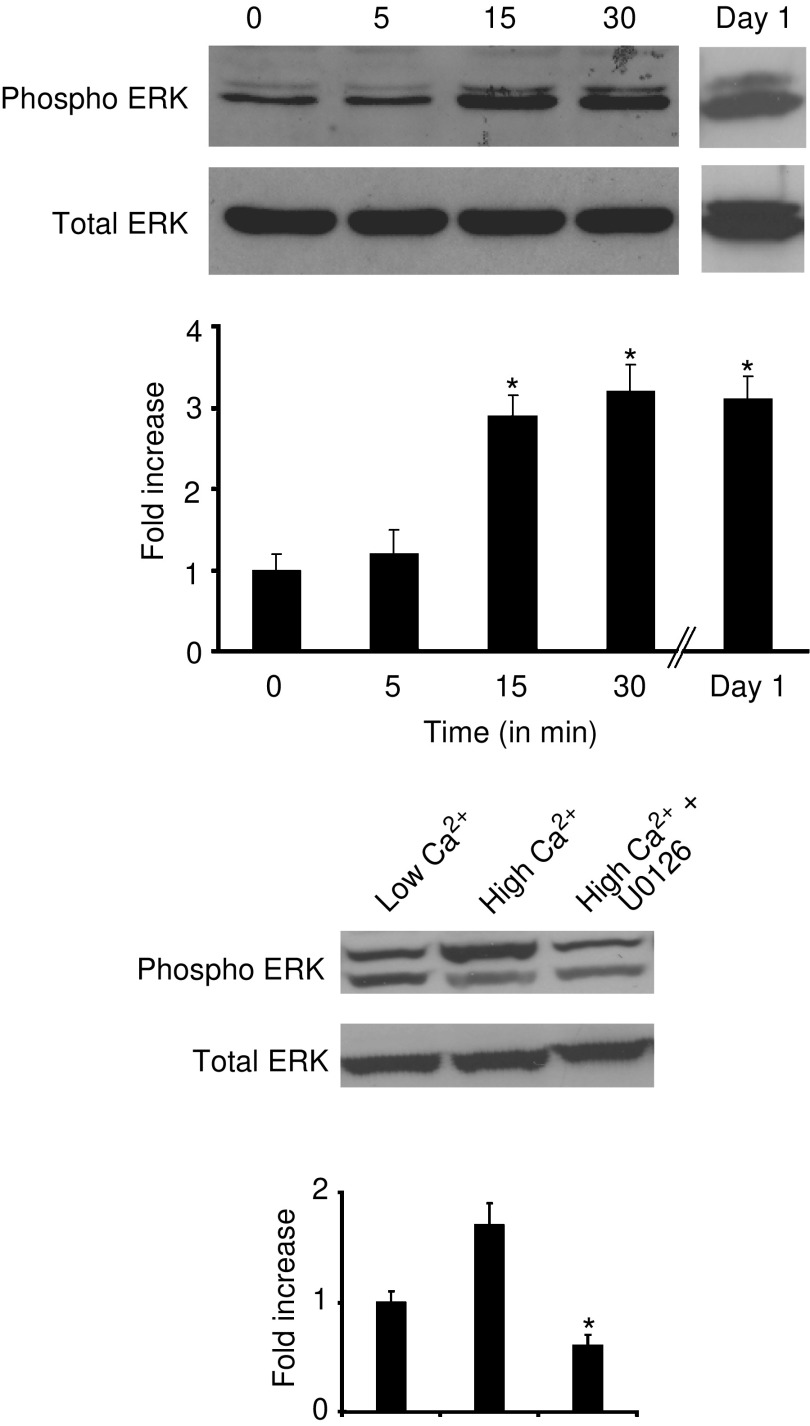
Effect of extracellular Ca^2+^ and U0126 on expression of phosphorylated-ERK. *Upper panel*: time-course for Ca^2+^-induced ERK phosphorylation. Lysates were prepared from CBS cells at various times after exposure to Ca^2+^ and analysed for phosphorylated-ERK by Western blotting. *Lower panel*: inhibition of Ca^2+^-induced ERK phosphorylation in the presence of U0126. CBS cells were grown for 1 day in Ca^2+^-free medium (left), in medium supplemented with 1.4 mM Ca^2+^ (centre) and in medium supplemented with 1.4 mM Ca^2+^ and 10 *μ*M U0126 (right). Lysates were prepared and analysed for ERK phosphorylation by Western blotting. Quantitative values are based on densitometry scanning of bands in four separate experiments and are means and s.d. Statistical significance was determined by ANOVA, followed by paired group comparisons. In *upper panel*, ^*^ indicates difference from zero-time value at *P*<0.05 level. In *lower panel*, ^*^ indicates differences from high-Ca^2+^ group (1.4 mM Ca^2+^ supplementation) at the *P*<0.05 level.

Next, CBS cells were incubated for 1 day in SMEM with dialysed FBS with or without Ca^2+^ and treated with U0126. Lysates were prepared from the cells at various times after treatment and probed for phosphorylated ERK and total ERK protein expression. As shown in Figure 6, there was no effect on ERK phosphorylation 5 min after exposure to extracellular Ca^2+^ but an increase was observed at 15 and 30 min post-treatment. ERK phosphorylation remained elevated through 1 day. When lysates prepared from cells treated with 10 *μ*M U0126 in Ca^2+^-supplemented medium were examined, ERK-phosphorylation was reduced to below basal level. Neither Ca^2+^-supplementation nor U0126 had a measurable effect on total ERK protein levels (Figure 6).

### Differences in Ca^2+^ sensitivity among cloned CBS isolates

**Figure 7:**
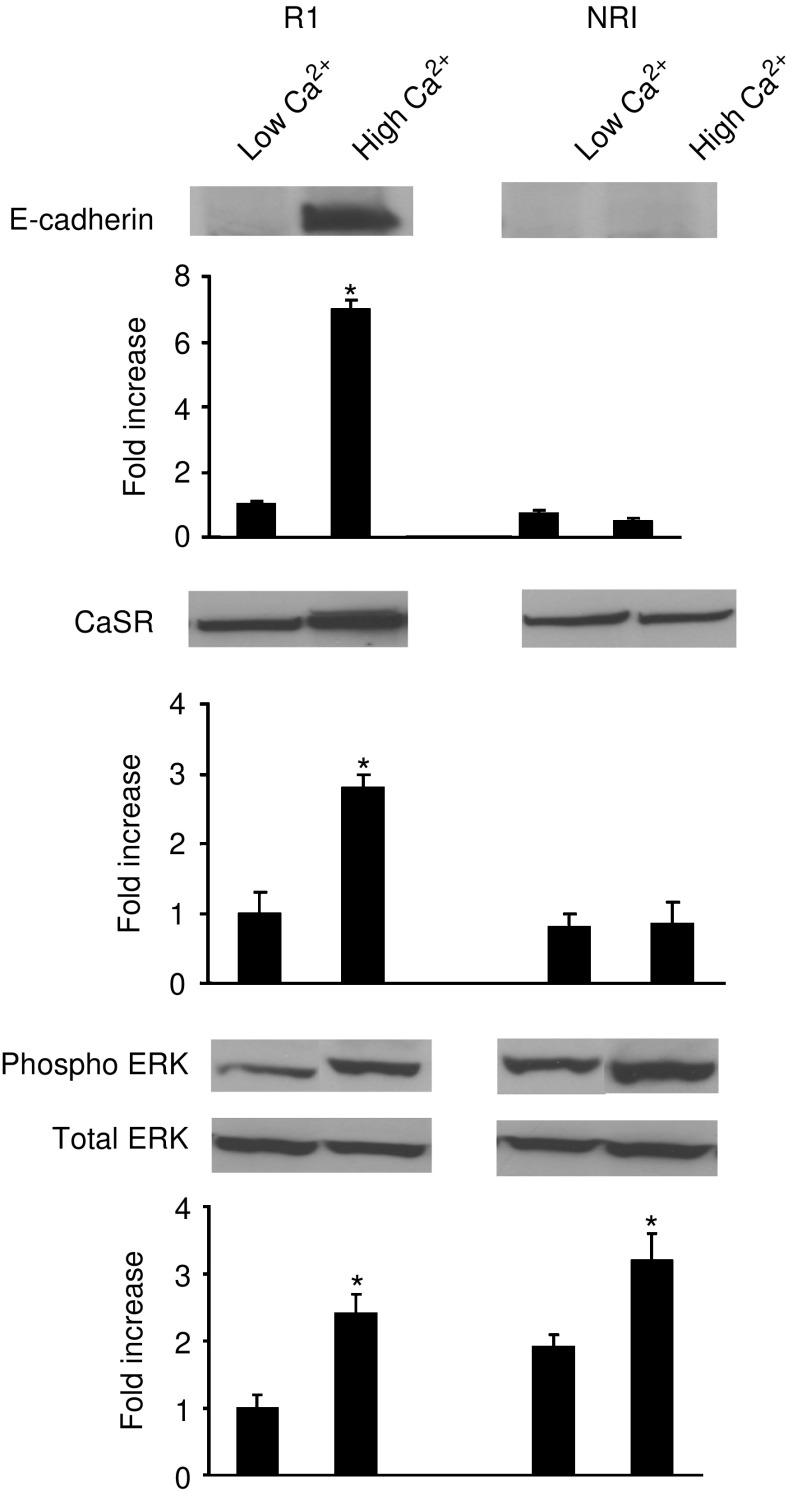
Effect of extracellular Ca^2+^ on E-Cadherin, CaSR, and phosphorylated-ERK expression in Ca^2+^-responsive and nonresponsive CBS clones. *Upper panel*: E-cadherin expression; *Middle panel*: CaSR expression; *Lower panel*: ERK expression. R1 and NR1 isolates of CBS cells were grown for three days in Ca^2+^-free medium (left) and in medium supplemented with 1.4 mM Ca^2+^ (right). Western blotting was used to assess the proteins. Quantitative values are based on densitometry scanning of bands in three separate experiments and are means and s.d. Statistical significance was determined by Student's *t*-test, comparing nonsupplemented and Ca^2+^-supplemented conditions for the two isolates separately. ^*^indicates differences from the nonsupplemented medium at the *P*<0.05 level.

In all, 12 separate isolates of randomly-cloned CBS cells were incubated in duplicate wells. One well of each duplicate was maintained in Ca^2+^-free SMEM (plus dialysed FBS) while the other was incubated in the same culture medium supplemented with extracellular Ca^2+^ to a final concentration of 1.4 mM. After 3 days, lysates were prepared from cells in each well and examined for E-cadherin expression by Western blotting. In none of the 12 isolates was there significant E-cadherin expression in the absence of Ca^2+^-supplementation. In Ca^2+^-supplemented medium, six of the isolates expressed high levels of E-cadherin while in the other six isolates there was little or no E-cadherin expression. Western blots from representative Ca^2+^-responsive and -nonresponsive isolates (termed R1 and NR1, respectively) are shown in Figure 7 (upper panel). Not surprising, the isolate that expressed E-cadherin in response to Ca^2+^-stimulation underwent morphological differentiation in Ca^2+^-supplemented medium and demonstrated reduced growth as compared to cells in nonsupplemented medium (46% inhibition; *n*=3; *P*<0.05 by Student's *t*-test). In contrast, the cells that did not express E-cadherin in response to Ca^2+^-stimulation failed to differentiate (morphologically) in Ca^2+^-supplemented medium and did not demonstrate a significant reduction in growth (4% reduction relative to nonsupplemented medium; *n*=3; ns by Student's *t*-test).

The same Ca^2+^-responsive isolate R1 and Ca^2+^-nonresponsive isolate NR1 were maintained for 3 days in SMEM plus dialyzed FBS with or without added Ca^2+^. At the end of the incubation period, lysates were prepared and examined for CaSR expression by Western blotting. Both isolates expressed detectable levels of CaSR. In nonsupplemented medium, the levels expressed by the two isolates were similar to the level expressed by the uncloned parent population (Figure 7, middle panel). In Ca^2+^-supplemented medium, however, differences were observed. Specifically, isolate R1 showed the same upregulation of CaSR as the parent cells (compare Figure 7, middle panel with Figure 5). In contrast, isolate NR1 failed to upregulate CaSR under the same conditions (Figure 7, middle panel).

In a final set of experiments, the phosphorylation pattern of ERK was assessed in R1 and NR1 isolates grown for 1 day in SMEM plus dialysed FBS with or without added Ca^2+^. As shown in the lower panel of Figure 7, there was a significant increase in ERK phosphorylation in Ca^2+^-supplemented medium in both isolates, as compared to what was seen in nonsupplemented medium. There were no differences in the total ERK protein levels between the two isolates under either condition. Not surprisingly, U0126 (10 *μ*M) suppressed levels of phosphorylated ERK to below baseline levels in both isolates in Ca^2+^-supplemented medium (not shown).

## DISCUSSION

Extracellular Ca^2+^ is a critical regulator of epithelial cell growth and differentiation, but the mechanistic events that lead from an increase in extracellular Ca^2+^ to growth inhibition and onset of differentiation are not fully understood. Induction of E-cadherin is known to be important. E-cadherin is minimally expressed in rapidly-growing epithelial cells *in vitro* but is upregulated during differentiation (Birchmeier 1995; Mareel *et al*, 1996). *In vivo*, the loss of E-cadherin from the cell surface occurs during tumour progression to a more malignant phenotype (Birchmeier 1995; Mareel *et al*, 1996; Van Aken *et al*, 2001). This occurs in conjunction with a loss of differentiated histological features. In recent studies, we demonstrated that Ca^2+^-induced growth arrest and differentiation in CBS colon carcinoma cells were associated with E-cadherin elaboration and, ultimately, with cell surface E-cadherin expression. Among the events associated with E-cadherin accumulation in the membrane was a reduction in intra-nuclear complexes of *β*-catenin/TCF4 (possibly due to competition of surface E-cadherin for *β*-catenin) and downregulation of TCF4 transcription. Additional events associated with Ca^2+^ treatment in CBS cells included increased production of the cell cycle inhibitors P21 and P27, increased *γ*-catenin expression and increased cytokeratin expression (Chakrabarty *et al*, 2003, 2005; Giles *et al*, 2003; Kamei *et al*, 2003). These changes, alone or in combination, may ultimately be responsible for the growth-suppression and onset of differentiation seen in the colon epithelial cells.

The present study provides evidence that MAP kinase signalling and CaSR upregulation are both critically involved as upstream regulators of Ca^2+^-induced E-cadherin expression (and the subsequent changes in growth and differentiation) in cells derived from human colon cancer. The two events appear to be, at least in part, independent of one another. Using a line of colon carcinoma cells, we found that cells equilibrated in Ca^2+^-free medium and then exposed to 1.4 mM Ca^2+^ underwent a (relatively) slow but sustained rise in phosphorylated ERK. The same conditions that led to a rise in ERK phosphorylation (activation) also resulted in expression of E-cadherin and with reduced growth and increased differentiation. U0126, a potent inhibitor of MEK-dependent ERK-activation, prevented Ca^2+^-induced E-cadherin expression. Concomitantly, the reduction in cell proliferation and onset of differentiation normally seen in Ca^2+^-supplemented medium did not occur. U0126 was effective at 10 *μ*M, a concentration at which the inhibitor is known to be highly specific for MEK-dependent ERK activation (Favata *et al*, 1998). Inhibitors of three other signalling pathways did not duplicate these effects.

The ERK family of MAP kinases consists of a cascade of intracellular signalling elements that respond to a number of extracellular stimuli, including soluble peptide growth factors and components of the extracellular matrix. Peptide growth factors induce a sharp rise in activation (20–50-fold over baseline). Activation tends to be rapid (peaks within 2–5 min) and transient, with the active kinases falling to some intermediate level within 15–30 min. Under such conditions, a proliferative response is seen (Zheng *et al*, 1994; Chen *et al*, 1996; Zeigler *et al*, 1999). MAP kinase activation following cell contact with the extracellular matrix (integrin-mediated) results in a smaller increase (in the range of 2–6-fold increase over baseline) but the response is sustained. MAP kinase activation in this manner is associated with a differentiation response rather than with proliferation (Marshall, 1995).

Only a relatively few previous studies have addressed the relationship between MAP kinase signalling and E-cadherin expression. The data are complex and seemingly inconsistent. In one recent study, a switch from low- to high- extracellular Ca^2+^ was shown to induce ERK activation in an immortalised epidermal keratinocyte line (Pece and Gutkind, 2000) – similar to what we have observed here. In that study, however, E-cadherin was purposed to be a critical intermediate in the MAP kinase activation sequence since disruption of E-cadherin function with an anti-E-cadherin antibody prevented the rise in MAP kinase signalling. In another study, epithelial cells were maintained under sparse conditions (undifferentiated state) or in dense culture (where differentiation occurred) and examined for ERK expression. Cells in sparse culture expressed high levels of phosphorylated ERK relative to what was seen in dense culture. In the presence of an ERK inhibitor (PD98059), levels of phosphorylated ERK were reduced. Concomitantly, E-cadherin expression was increased (Conacci-Sorrell *et al*, 2003). Based on this, it was concluded that ERK activation was responsible for suppression of E-cadherin, and for preventing differentiation in sparse culture. In yet another study, MAP kinase blockade was found to suppress growth of colon tumours *in vivo* (Sebolt-Leopold *et al*, 1999). Presumably, growth suppression *in vivo* reflected interference with signals generated via interaction of cell surface receptors with peptide growth factors. While our data do not conflict, *a priori*, with these previous observations, it is clear from the present study that in the CBS colon carcinoma cells, MAP kinase activation is a critical component in the sequence of events that leads from extracellular Ca^2+^ stimulation to the induction of E-cadherin, and, ultimately, to growth suppression and onset of differentiation. Based on the previous findings of others (reviewed in Alpin *et al*, 1998), we can suggest that interaction of CBS colon carcinoma cells with components of the extracellular matrix constitutes the initial stimulus for ERK activation and the subsequent downstream events (including, presumably, E-cadherin expression) that follow. While direct evidence to support this hypothesis is lacking, what is clear is that in CBS cells, ERK activation is upstream in the sequence of events that lead from Ca^2+^ stimulation to growth arrest and differentiation.

Induction of CaSR appears to be a second critical event leading from Ca^2+^ stimulation to growth arrest and differentiation. This is based on the observation that in the mixed population of parental CBS cells, there are cells that do not respond to Ca^2+^ stimulation with the expected induction of E-cadherin or in growth-retardation. When these cells were isolated and cloned, they also failed to demonstrate upregulation of CaSR in response to Ca^2+^ stimulation. How Ca^2+^ stimulation leads to CaSR induction is not fully understood. The CaSR gene has promoter regions that are sensitive to both Ca^2+^ and vitamin D (Canaff and Hendy 2002). Response elements for the two agonists are different, such that a mutation in one site can affect binding of one agonist without affecting the other (Chakrabarty *et al*, 2005). Studies are underway at present to determine if clones of CBS cells that do not undergo growth arrest or differentiation in response to Ca^2+^ stimulation have defects in the Ca^2+^-binding region of the CaSR promoter. If Ca^2+^ is a direct activator of CaSR, one would predict (consistent with what we have observed) that downstream signalling events would not be required for induction. Additional studies will be needed to verify this suggestion and to fully explore the mechanism of Ca^2+^-induced CaSR expression.

The findings in the present report are based on studies in a single line of moderately-differentiated human colon carcinoma cells. However, our findings are probably not unique to these cells. In a recent study, we showed that in four different human colon carcinoma cell lines (including CBS), raising the extracellular Ca^2+^ concentration resulted in induction of E-cadherin and inhibition of growth (Chakrabarty *et al*, 2003). A direct relationship between CaSR expression and the differentiated phenotype has also been observed in both the normal colonic mucosa and malignant colonic epithelium (Sheinin *et al*, 2000; Chakrabarty *et al*, 2003, 2005).
